# Getting Rid of Patient’s Misconceptions About the Radiology Department Using Animated Video in the Waiting Room

**DOI:** 10.5334/jbsr.2405

**Published:** 2021-07-29

**Authors:** Michel Lavaerts, Hilde Vandenhout, Raymond Oyen, Chantal Van Ongeval

**Affiliations:** 1 UZLeuven, BE

**Keywords:** Radiology, Learning-curve, Animated Video, Waiting Room, Misconception, Education

## Abstract

**Objectives:**

Patients often confuse the role of the radiologist with that of the technician. The aim of this study is to explore patients’ current perceptions about the radiology department and to evaluate how it’s possible to get rid of misconceptions using informative animated video in the waiting room.

**Materials and Methods:**

In this multi-centric study (UZ Leuven, ZNA Middelheim), 278 patients of all ages and education levels were surveyed in the radiology waiting room. 107 patients filled out the survey after watching an informative animated video (*www.makeradiologyvisible.com*). The remaining patients did not watch the video.

**Results:**

Half of the patients (86/171) in the non-video group believe the radiologist “performs the scanning”, compared to 19% (20/107) in the video group (p < 0.001). Patients who think their own physician will interpret the images is 36% (61/171) in the non-video group and 10% (11/107) in the video group (p < 0.001). In the non-video group, 32% (55/171) believe the technician performs the exam compared to 59% (63/107) in the video group (p < 0.001). After the video, 67% (72/107) of patients have more respect for the work of the radiologist, 52% (56/107) experience less anxiety and 65% (70/107) think the video is of added value to their visit. All items showed a better impact in high-educational subgroups.

**Conclusion:**

Animated informative videos help to increase patient knowledge about the radiology department. It moderates expectations, reduces anxiety, and ameliorates the overall experience. Although, the learning curve is steeper in highly educated patients, all educational levels benefit.

## Introduction

Throughout history, the diagnostic radiologist has developed an increasing distance from the patient [1]. Because of modern imaging techniques and higher workloads, radiologists today seldom get to meet their patients. Besides, there are numerous popular medical TV shows in which a radiologist rarely is involved, which does not improve the overall perception of their role [2]. In 2012, a study in the British Journal of Radiology reported that 76% of patients surveyed in a breast radiology waiting room think the radiologist “takes the x-rays”, 86% say radiologists are “not doctors” and 40% believe radiologists “play no role in patient care” [3]. A study by Grant et al. found that up to 38% of patients do not consider radiologists as part of the medical team [4]. Hence, there is a significant lack of awareness amongst patients regarding the role and responsibilities of the modern diagnostic radiologist.

One can expect that patients will better comply to medical advice if the medical qualifications of the radiologist are well understood. Likewise, patient care (or the perception of care) could be impacted because the radiologist’s medical qualifications are misunderstood [5]. Besides, the public’s perception of physicians in general (at least in the industrial world) has changed from profound respect, towards physicians being seen more as business minded. Newspaper publications of average incomes for specialists do not soften that image [6]. It is thus imperative for radiologists to educate the public and increase awareness of the radiology profession so that future changes in the health service will reflect the scope and importance of radiology.

We hypothesized that an informational animated video could help educate patients in the radiology waiting room about the specific role of the radiologist in their care. We created a two-minute animated video in which we walk the patient through the radiology process. In this study, we investigated the impact of such a video on patient knowledge about the job of radiologist/technician. We examined whether the learning curve depended on patient educational levels.

## Materials and Methods

### Study design

**Figure 1:**
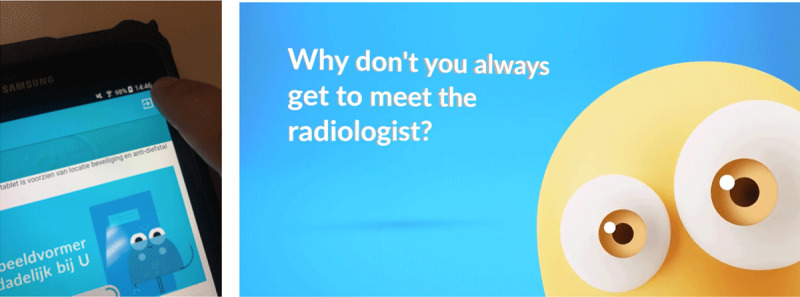
Survey was taken on tablets in the waiting room. Snapshot of the informative animated video that was playing in the radiology waiting rooms.

A total of 278 patients (122 men, 156 women) were surveyed in our study conducted in two different hospitals in Belgium (UZ Leuven, ZNA Middelheim). Every patient who presented to the Magnetic Resonance Imaging (MRI), Computed Tomography (CT) and X-ray waiting rooms, willing and able to fill out the survey was included. The inclusion criterion was an average knowledge of the Dutch language in order to understand all questions. The survey was filled out on portable tablets to reach every age group. In our experience, all ages feel more comfortable with this technology compared to computers (***Figure 1***). The survey was not conducted in ultrasound waiting rooms as these exams are performed by the radiologist in Belgium. The goal of the study is to teach patients a basic understanding of who performs medical exams (the technician) and who interprets the images (the radiologist).

First, patients were surveyed without a video playing. On a different day, a non-intrusive, muted, animated, informative video was shown on a wall-mounted TV in loop, in the radiology waiting room. In the video, are explained the consecutive steps patients go through when having an imaging exam. We explain that the patient will be called out by a technician to the exam room. Afterwards, technicians will position and guide the patient during the exam and produce all the images in close contact with the supervising radiologist. Patients are told they would ask the technician to meet the supervising radiologist if necessary since, they will not likely need to meet him otherwise. After the exam, all data are sent to the diagnostic radiologist who will interpret the images, make a diagnosis, and issue a report. The video explains that a radiologist is a certified medical doctor with an additional five-year specialization in medical imaging interpretation after the master’s degree in medicine. Lastly, a short fragment of the video teaches patients about the work behind the scenes as the diagnostic radiologist interacts with other medical specialists that are also interventional radiologists. (See the video here or on *www.makeradiologyvisible.com*.)

Every patient was asked at the beginning of the survey “did you see the video play?”, which allows us to divide those surveyed in two groups: A baseline “non-video group” which totalled 171 patients (61.5%) and a “video group” of 107 patients (38.5%). Why some patients did not see the video when it was playing was beyond the scope and the intent of this study. Plausible reasons are smartphone usage and short radiology waiting times. Nobody was actively asked to look at the video. The video group was additionally questioned on their appreciation of the video and what influence it had on their visit.

The data collection was anonymous and only non-specific demographic data were obtained (sex, age-group, highest educational level, nationality). Multiple-choice questionnaire in the survey included the following:

– Did you see the animated video play?
– Who performs the exam?
– What is the qualification of the radiologist?
– What are the core tasks of the radiologist?
– How long (after high school) does it take to become a radiologist?
– What education does a radiologist have?
– What is the role of the radiologist in your care?
– Do you expect to meet the radiologist?

Patients who watched the video received those additional questions:

– Were you more at ease prior to the exam, because of the video?
– Was the video of added value for your visit?
– Do you feel more respect for the job of the radiologist after watching the video?

Ethical evaluation and approval were obtained from the OBC of the Catholic University of Leuven, Belgium (File number: MP015121). To measure the changes in patient understanding about the role of the diagnostic radiologist, results were computed and analysed using Qualtrics. Proportions are given in percentages and compared using chi-squared tests.

## Results

In the non-video group, 43% (73/171) of patients select “taking X-rays” and 50% (86/171) select “performing scans” as core tasks for the radiologist. In the video group, that number lowers respectively to 14% (15/107) and 19% (20/107) (all p-values < 0.05). Only half of the patients (50%, 86/171) in the non-video group know that radiologists have a “full medical degree”, whereas the video group patients realise radiologists are doctors in a higher frequency (72%, 77/107, p < 0.001).

Further, in the non-video group, 66% (113/171) patients know that part of the radiologist’s job is to interpret the images and report the findings, whereas in the video group that number rises up to 94% (101/107, p < 0.001). Only 32% (55/171) know that the exam will be conducted by technicians, and in the video group this number almost doubles to 59% (63/107, p < 0.001). 36% (61/171) of patients in the non-video group believe the referring physician does the imaging interpretation and in the video group this frequency drops to a mere 10% (11/107, p < 0.001). In the non-video group, 47% (81/171) expect to meet the radiologist compared to 30% (32/107, p < 0.01) in the video group (***Table 1***).

**Table 1:**
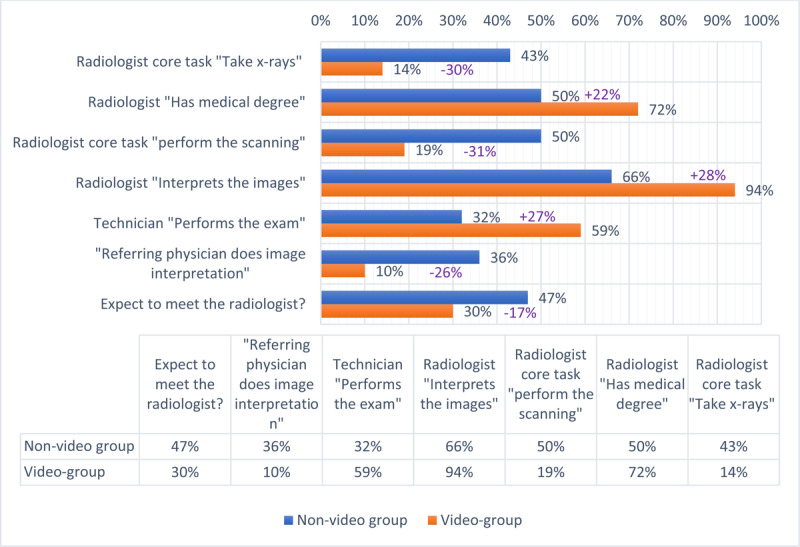
Better patient perception of the role of the radiologist, non-video (blue) versus video-group (orange).

### IMPACT of Patient Educational Level

Out of 278 patients, 243 provided information about their educational level; 53% (142) of patients had a maximum educational level of a high school diploma (“the less-schooled group”); 38% (101) had a higher degree of education (“the well-schooled group”). Both educational groups show a similar percentage of patients in the video-group (***Table 2***).

**Table 2:** Patients who provided their educational level were split in two groups to evaluate learning curves based on the information shown in the video. A cut-off was made at the high school degree: the “less-schooled” (max high-school degree) and the “well-schooled” (higher education).


	TOTAL (N = 243, 100%)				GROWTH LESS-SCHOOLED	GROWTH WELL-SCHOOLED

	LESS-SCHOOLED (N = 142, 58%)		WELL-SCHOOLED (101, 42%)			
	
	NON-VIDEO GROUP (N = 87, 61%)	VIDEO GROUP (N = 55, 39%)	NON-VIDEO GROUP (N = 61, 60%)	VIDEO GROUP (N = 40, 40%)		

**1. Correct answer to: Radiologist qualification**	30 (34.5%)	24 (43.6%)	24 (39.3%)	28 (70.0%)	+9.1%	+30.7%

**2. Correct answer to: Radiologist degree**	30 (34.5%)	32 (58.2%)	37 (60.6%)	37 (92.5%)	+23.7%	+31.9%

**3. Correct answer: Technician makes the images**	22 (25.3%)	31 (56.4%)	21 (34.4%)	26 (65.0%)	+31.1%	+30.6%

**4. Correct answer to: Duration radiology education**	5 (5.7%)	14 (25.5%)	6 (9.8%)	25 (62.5%)	+19.8%	+52.7%

**5. Correct answer to: what’s the radiologist’s role?**	46 (52.9%)	45 (81.8%)	37 (60.7%)	38 (95.0%)	+28.9%	+34.3%

**Total growth in both groups:**					**+22.5%**	**+36.0%**


**Figure 2:**
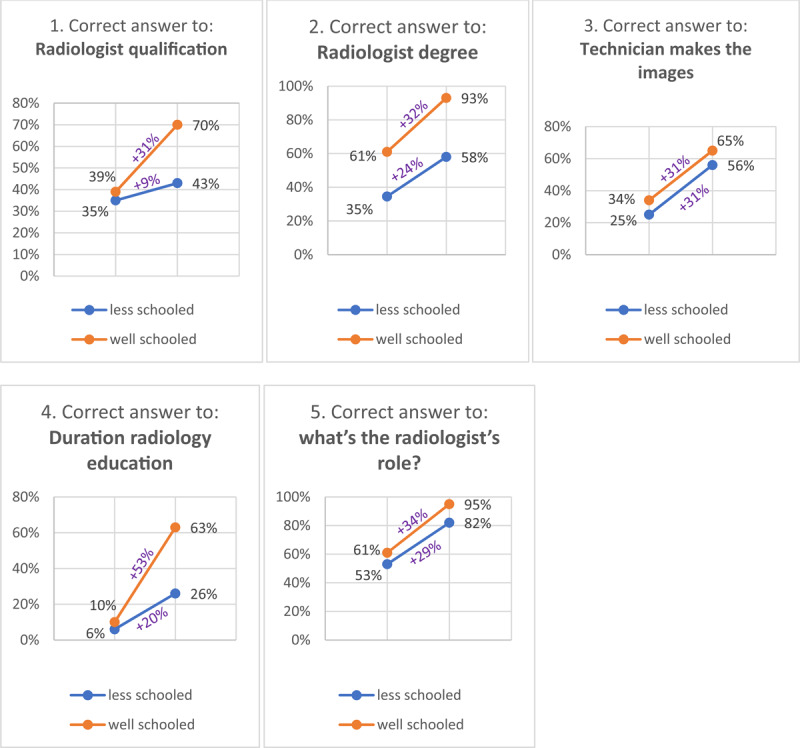
Learning curves in table 2 visualized (cut-off at between “less-” and “well-schooled” is possession of a high-school diploma).

In the non-video group, 35% (30/87) of the less-schooled patients are correctly informed about the medical qualifications of the radiologist, compared to 39% (24/61) in the well-schooled group. After watching the video, 43% (24/55) of the less-schooled (+9%; p = 0.34) and 70% (28/40) of the well-schooled (+31%; p = 0.0024) patients answered correct (***Figure 2***).

When the non-video group is questioned on how long their radiologist has studied after high school, only 6% (5/87) of the less-schooled check the “>10 years” box, compared to 10% (6/61) in the well-schooled group. After seeing the video, 26% (14/55, p < 0.001) of the less-schooled group answers correctly, a moderate increase. The well-schooled group now answers correctly in 63% (25/40, p < 0.001) of cases. Again a steeper increase (+53% compared to +16% in the less-schooled group).

When asked if the radiologist has a master’s degree in medicine, 45% (67/148) in the non-video group answers correctly, in the video group 73% (69/95) answers correctly (p < 0.001), this equals +32% in the well-schooled group compared to +18% in the less-schooled group.

### The Video Group In-Depth

**Figure 3:**
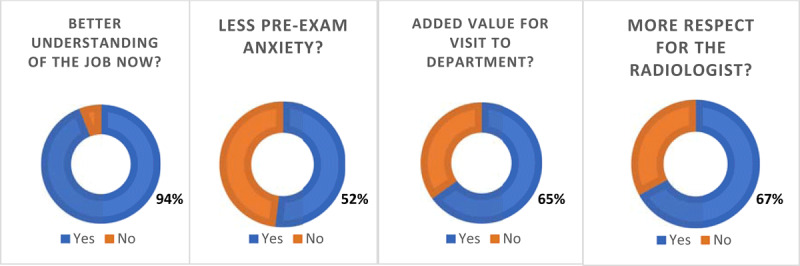
Extra questions for the “video group”.

The video group is further evaluated on the use of method for information transfer (animated video). Almost all 94% (100/107) perceives a better understanding of what a radiologist is and does. Nearly half of patients 52% (56/107) have less pre-exam anxiety and feel more at ease in the waiting room. Most patients 65% (70/107) reacted fondly on the extra information and the format in which it was presented, for them it was of added value for their visit. Most strikingly, 67% (72/107) of the patients feel more respect towards the radiologist solely due to the video/obtained knowledge, without having to meet a radiologist (***Figure 3***).

## Discussion

In the past, several communication methods have been used to educate patients on the role of the radiologist. For example, Madden et al. started to inform breast patients pre-exam through booklets, which greatly improved the levels of knowledge [7]. In 2019, several web-based platforms were studied for patient-centred communication in our current digital age. This study emphasized once again why communication is paramount to the patient-centred imaging experience of today [8].

However, as radiologists we must feel obliged to search for an alternative-modern way of communicating to maintain a connection with our patients without increasing workload and without risking changing the patient-doctor relationship. Short, fun-to-watch, animated videos are ubiquitous and represent a superb tool to capture the attention of all ages. They have the advantage of being easy, cost effective, environmentally- friendly tools to transfer information to masses without being intrusive. Animated videos are known for a better short and long-term retention of information compared to non-animated graphical information [9,10,11,12]. However, the advantage over well-designed static materials in patients with low health literacy shows no objective significance [13]. This communication method has proven to be easy to understand, practical, and leads to a better patient waiting experience while decreasing anxiety. The 52% decrease in pre-exam anxiety in our study is in line with results found in literature [14,15,16,17]. Even though the perceived waiting room time is not significantly shortened when using animated video, waiting satisfaction scores are significantly higher [18].

For the above reasons we opted to produce a modern animated *video* to educate patients on how the radiology department works and what they can expect from their visit while waiting in the radiology waiting room (*www.makeradiologyvisible.com*). When compared the study of O’Mahony et al. [3], Belgian patients seem more aware of what a radiologist and a technician are, although the knowledge of the job content is still somehow low. Overall, well-schooled patients have a better perception of the jobs of the radiologist and technician compared to the less-schooled group, prior to seeing the video. Even though baseline knowledge already is better in the well-schooled group, the gain in knowledge in the video group is significantly higher in the well-schooled group (average +36%) compared to the less-schooled group (average +22%). However, the improvement of patient perception of radiology jobs, after seeing the video, was high on all educational levels. Thus, all patient educational levels benefit from informational animated video although the learning curve is steeper when patients have a higher degree of education.

**Video:** ENGLISH.

**Video:** DUTCH.

Caution is needed when comparing our results with the study of O’Mahony et al. [3] because that was held in the breast radiology waiting room while our study was held in MRI/CT and X-ray waiting rooms. Importantly, the breast radiologist does often meet the patient, contrary to the modality waiting rooms we have chosen to perform our study.

It is also plausible that our patients have a better understanding of the role of the radiologist compared to those in the UK where the technician is named “radiographer” which could be confusing given this term similarities with the word “radiologist”. The Dutch word for a technician meanwhile is a literal translation of “image-maker” that quite obviously explains the job content and therefore causes less confusion.

Different patients were surveyed in the non-video versus the video group (all were surveyed prior to their exam). Therefore, we cannot objectify a person-based learning curve. Population-wide interpretation of the results can be justified given the consistent outcomes in both institutions (that also showed similar demographics).

## Conclusion

According to this study, animated informative video in the radiology waiting room helps getting rid of misconceptions about the role of the radiologist and the technician in all educational levels, although well-schooled patients benefit more. Animated informative video is of added value for a radiology department visit as it decreases pre-exam anxiety and increases overall patient respect for the radiology profession, without having to meet a radiologist in person.
